# Maternal anaemia and duration of zidovudine in antiretroviral regimens for preventing mother-to-child transmission: a randomized trial in three African countries

**DOI:** 10.1186/1471-2334-13-522

**Published:** 2013-11-06

**Authors:** Benn KD Sartorius, Matthew F Chersich, Mary Mwaura, Nicolas Meda, Marleen Temmerman, Marie Louise Newell, Timothy MM Farley, Stanley Luchters

**Affiliations:** 1 School of Public Health, Faculty of Health Sciences, University of the Witwatersrand, Johannesburg, South Africa; 2 Discipline of Public Health Medicine, School of Nursing and Public Health, University of KwaZulu-Natal, Mtubatuba, South Africa; 3 International Centre for Reproductive Health, Department of Obstetrics and Gynaecology, Ghent University, Ghent, Belgium; 4 International Centre for Reproductive Health, Mombasa, Kenya; 5 Centre Muraz, Bobo Dioulasso, Burkina Faso; 6 Africa Centre for Health and Population Studies, University of KwaZulu-Natal, Mtubatuba, KwaZulu-Natal, South Africa; 7 Sigma3 Services SARL, Nyon, Switzerland; 8 Centre for International Health, Burnet Institute, 85 Commercial Road, Melbourne, Victoria 3004, Australia

**Keywords:** Zidovudine, Pregnancy, HIV, Sub-Saharan Africa, Anaemia, Drug toxicity

## Abstract

**Background:**

Although substantiated by little evidence, concerns about zidovudine-related anaemia in pregnancy have influenced antiretroviral (ARV) regimen choice for preventing mother-to-child transmission of HIV-1, especially in settings where anaemia is common.

**Methods:**

Eligible HIV-infected pregnant women in Burkina Faso, Kenya and South Africa were followed from 28 weeks of pregnancy until 12–24 months after delivery (n = 1070). Women with a CD4 count of 200-500cells/mm^3^ and gestational age 28–36 weeks were randomly assigned to zidovudine-containing triple-ARV prophylaxis continued during breastfeeding up to 6-months, or to zidovudine during pregnancy plus single-dose nevirapine (sd-NVP) at labour. Additionally, two cohorts were established, women with CD4 counts: <200 cells/mm^3^ initiated antiretroviral therapy, and >500 cells/mm^3^ received zidovudine during pregnancy plus sd-NVP at labour. Mild (haemoglobin 8.0-10.9 g/dl) and severe anaemia (haemoglobin < 8.0 g/dl) occurrence were assessed across study arms, using Kaplan-Meier and multivariable Cox proportional hazards models.

**Results:**

At enrolment (corresponded to a median 32 weeks gestation), median haemoglobin was 10.3 g/dl (IQR = 9.2-11.1). Severe anaemia occurred subsequently in 194 (18.1%) women, mostly in those with low baseline haemoglobin, lowest socio-economic category, advanced HIV disease, prolonged breastfeeding (≥6 months) and shorter ARV exposure. Severe anaemia incidence was similar in the randomized arms (equivalence *P*-value = 0.32). After 1–2 months of ARV’s, severe anaemia was significantly reduced in all groups, though remained highest in the low CD4 cohort.

**Conclusions:**

Severe anaemia occurs at a similar rate in women receiving longer triple zidovudine-containing regimens or shorter prophylaxis. Pregnant women with pre-existing anaemia and advanced HIV disease require close monitoring.

**Trial registration number:**

ISRCTN71468401

## Background

Anaemia during pregnancy and lactation is common, affecting ~40% of women globally [1,2]. Highest levels of anaemia in pregnancy occur in sub-Saharan Africa (SSA), where an estimated 57% (~17 million) of all pregnant women are anaemic (haemoglobin [Hb] < 11 g/dl) [3-6]. There is, however, marked variation in anaemia prevalence among pregnant women across the continent, ranging from 41-83%. Anaemia levels are highest in malaria-endemic areas [7], especially among the estimated one million pregnant women each year who are co-infected with malaria and HIV [7,8]. HIV infection itself, as with other chronic conditions, can cause anaemia [9]. A study among women attending antenatal clinics in Kenya, found as many as two-thirds of HIV-infected women were anaemic [10].

In addition to malaria and HIV, other factors account for high levels of anaemia in Africa, such as under-nutrition and iron deficiency, intestinal helminth infestation and higher pregnancy rates [11]. Anaemia in pregnancy increases risk of maternal mortality [12]. For each 1 g/dL decrease in mean Hb in late pregnancy, maternal mortality risk rises 20% (95% CI = 9-30%) [13]. Pregnant women with anaemia require faster and more vigorous intervention during pregnancy, delivery or postpartum should excessive bleeding occur [14-16].

The antiretroviral (ARV) zidovudine (ZDV) affects bone marrow function and can cause or exacerbate pre-existing anaemia, in addition to its classical manifestation of macrocytosis [17-20]. Concerns are heightened with use of this drug in pregnant women in SSA, given high anaemia levels and the limited capacity of health systems to respond to its consequences in pregnancy.

Previous randomised trials have examined the haematological safety of ZDV prophylaxis for preventing mother-to-child transmission (PMTCT). In these studies, ZDV was taken only during pregnancy and labour for up to 12 weeks. A randomised trial in Thailand, which compared women taking ZDV from 36 weeks of pregnancy with those on placebo, found similar haematocrit^a^ at delivery (32% *vs* 33%). Thirteen women receiving ZDV had severe postpartum anaemia (haematocrit < 25%), compared with 10 controls [21]. No major differences were seen in a subsequent study in Thailand [22]. In Cote d’Ivoire, with a similar PMTCT regimen, no differences in Hb concentrations were noted in women receiving ZDV compared with placebo [23,24]. A recent PMTCT trial in Malawi also did not find increased anaemia when comparing a longer (28 week) and shorter (1 week) ARV regimen [25], while by contrast other cohort studies in Malawi and Mozambique found a higher risk of anaemia in women taking a ZDV-containing regimen compared with a stavudine-based triple ARV regimen [26]. Further, more definitive, evidence of effects of current ARV regimens could inform policy and care for HIV-infected pregnant women.

This study describes haematological changes among women receiving ZDV-containing ARV regimens of varying duration in Burkina Faso, Kenya and South Africa. We assessed whether severe anaemia occurred more commonly among women randomized to longer ZDV-containing triple ARV prophylaxis in pregnancy and continued during breastfeeding, compared to a shorter regimen of ZDV in pregnancy and 1 week postpartum only. We also identified sub-groups at risk for severe anaemia.

## Methods

Data from the Kesho Bora trial in Burkina Faso, Kenya and South Africa were used. Detailed objectives, trial methods and primary endpoint analyses are presented elsewhere [27-30]. Pregnant women were enrolled from five sites in Burkina Faso, Kenya and South Africa (recruited at antenatal clinics) at 28–36 weeks gestation (or earlier depending on HIV disease stage and time of entry into study) and screened for eligibility after signing informed consent. To be eligible, HIV-infected women had to reside and plan to continue living in the study area until two years post-delivery, have no contraindication to receive ARVs, and no evidence of clinically significant conditions (obstetric, cardiac, respiratory including active tuberculosis, hepatic, gastrointestinal, endocrine, renal, hematologic, psychiatric, neurologic, or allergic) which may interfere with study interventions. Fifteen women with severe anaemia (Hb < 7.0 g/dL) at screening and who did not respond to medical management were excluded from study participation. Eligible women signed informed consent for study participation. Relevant ethical approval for study procedures were obtained [27].

HIV-infected pregnant women with WHO clinical stage 4 or a CD4 cell count below 200 cells/mm^3^ were enrolled in a prospective cohort (Cohort 1) and offered long-term ARV treatment (ZDV, lamivudine [3TC] and nevirapine [NVP]) beginning as early as possible in pregnancy and continued thereafter. Women with a CD4 >500 cells/mm^3^ were enrolled in a prospective cohort (Cohort 2) and offered a short-course regimen as per WHO recommendations [2,31]: 300 mg ZDV taken twice daily starting from 34 to 36 weeks of pregnancy until the onset of labour, plus one 600 mg dose of ZDV and 200 mg single dose (sd) of NVP at the onset of labour. These prospective cohorts were not initiated in South Africa. Women with intermediate immune status (CD4 count 200–500 cells/mm^3^) were randomised to receive an ARV prophylaxis regimen initiated between 28–36 weeks gestation, of either triple ARV prophylaxis (ZDV [300 mg], 3TC [150 mg], Lopinavir/ritonavir [LPV/r 400 mg/100 mg]) continued through delivery and during breastfeeding up to 6 months postpartum (RCT arm A); or the same short-course prophylaxis regimen used in Cohort 2 (RCT arm B) based on available WHO guidelines at the time from 2004 [31]. All infants received sd-NVP [2 mg per kg body weight] within 72 hours of birth. About midway through the trial (December 2006), following an update in WHO recommendations [2], women assigned the short-ARV regimen were also given seven days of ZDV/3TC after delivery and all infants received seven days of ZDV [4 mg per kg body weight] from birth, to reduce occurrence of ARV drug resistance.

Women visited antenatal clinic every two weeks from enrolment until delivery, following which mothers and their babies attended clinic at two, four, six, and eight weeks after delivery and then monthly until one year, and every three months thereafter until 24 months [27]. Due to fluctuations in available resources, the study protocol was altered in May 2006, restricting the follow-up duration to 12 months post-delivery, but then extended to 18 months from January 2008 onwards [27].

Using face-to-face structured interviews, socio-demographic data were collected at enrolment and information on variables such as infant feeding pattern were gathered at each scheduled visit. Blood samples were taken and a full blood count performed at: enrolment, 1 and 3 weeks after initiating the ARV regimen; delivery; weeks 2 and 6 postpartum, and at months 3, 6, 9, 12 and 18 after delivery. Hb testing was also done at unscheduled visits, where clinically indicated. This analysis utilized Hb measures from both scheduled and unscheduled visits.

Participants received a basic care package during pregnancy, including iron and folate (1 tablet of 60 mg iron plus 400 μg folate once daily for anaemia prevention, or twice daily for anaemia treatment), multivitamins once daily, presumptive treatment for malaria in endemic areas (once during third trimester), and anti-helminthics as recommended locally [27].

Individual ARV drugs causing toxicity were discontinued without necessarily discontinuing the whole regimen. If toxicity resolved, the drug could be restarted within 14 days. Full details of ARV options if permanent discontinuation was necessary are described elsewhere [27].

### Data analysis

**Figure 1:**
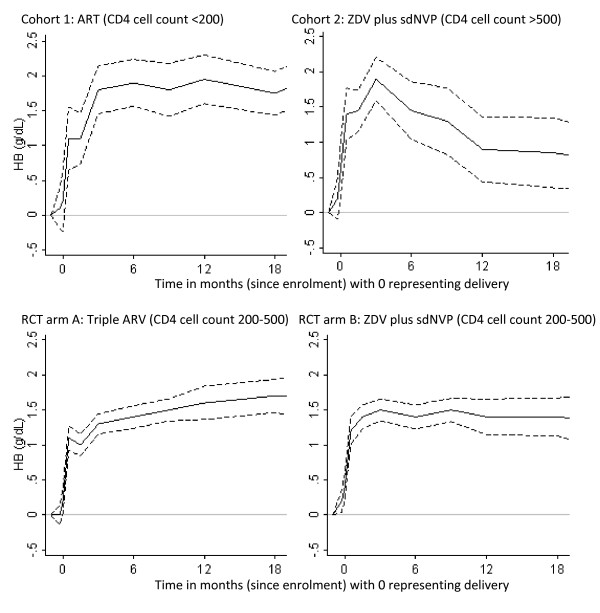
Median change (with 95% confidence intervals) in Hb level in the months before and after delivery (t = 0) by intervention arm.

Data were analysed using Stata 12.1 [32] and R [33]. Differences in Hb levels and occurrence of anaemia at enrolment and subsequent follow-up visits were compared among the cohort and trial arms using a Student’s *t*/Analysis of variance (ANOVA) test and Chi-square test respectively. We also used these tests to compare Hb and related parameters such as mean cell volume [MCV], mean cell Hb [MCH], haematocrit [HCT] and CD4 cell count at follow-up time points. An MCV above 100 fl was defined as macrocytosis [34]. For each woman, we also calculated the change in Hb at each follow-up point, compared to her enrolment Hb. The median of these values was computed (plus 95% confidence intervals) at each scheduled follow-up point, and compared between the cohorts and RCT arms (Figure 1).

**Figure 2:**
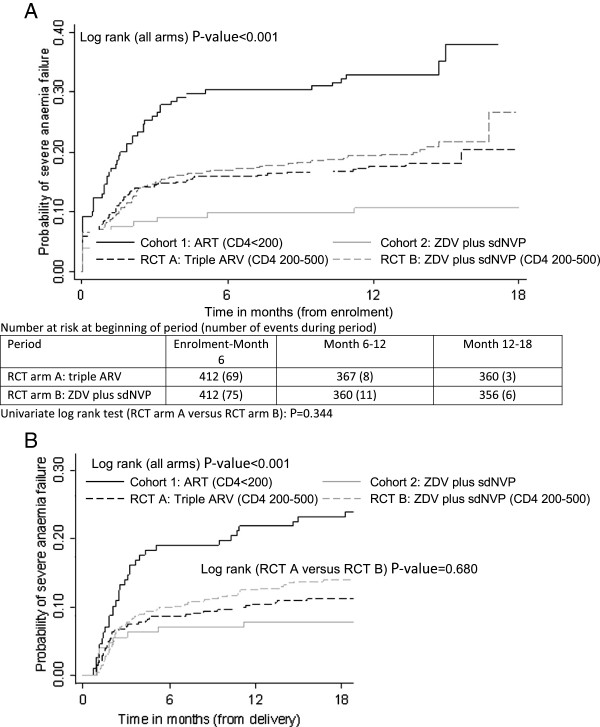
**Kaplan-Meier failure curve showing anaemia failure (Hb <8.0 g/dL) by intervention arm, with onset of risk at enrolment (Figure**2**A) and at delivery (Figure**2**B), censored at 18 months follow-up.**

**Figure 3:**
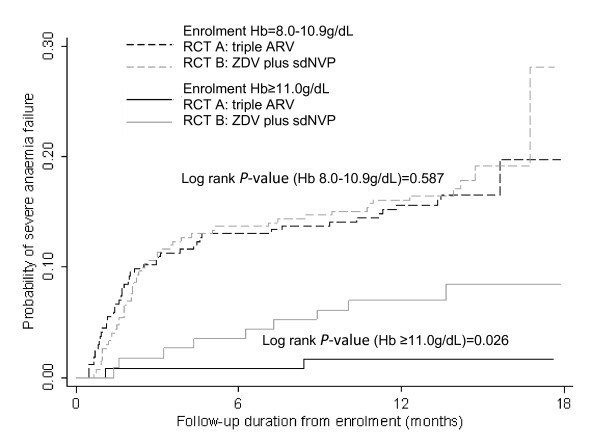
Kaplan-Meier failure curve showing anaemia failure (Hb < 8.0 g/dL) in RCT intervention arms, stratified by enrolment Hb level.

The primary outcome is occurrence of severe anaemia (defined as Hb < 8.0 g/dL) from study enrolment until 18 months follow-up, comparing the two RCT arms (and the two cohorts). Kaplan-Meier product-limit estimates were computed and log rank tests used to compare incidence of severe anaemia by cohort and RCT arm (Figure 2) and stratified by Hb level at enrolment for RCT arms only (Figure 3).

The aetiology of anaemia in pregnancy, especially in SSA, is complex and multi-factorial [35,36]. The following explanatory variables were considered when assessing the primary endpoint in bivariate and multivariable analysis: country, socio-demographic characteristics (age and education) and socio-economic status (SES) score at enrolment, body mass index [BMI], parity [first pregnancy versus 1 or more previous pregnancies], ever breastfed and prolonged duration of breastfeeding [none, <6 months, ≥6 months], advanced WHO HIV disease stage [classified as 3 or 4], and time-varying duration of ZDV use. SES was calculated using a weighted score from a principal component factor analysis based on eight household assets and divided into quintiles within each country.

The risk factor analyses were split into individual cohorts and RCT trial arms combined given the differences in the observational cohorts and absence of cohorts in South Africa. Women with severe anaemia were not considered at risk of another diagnosis of severe anaemia unless their Hb level returned to a value ≥8 g/dl. Variables significantly associated with severe anaemia at the 10% level were selected for inclusion into the various multivariable Cox non-parametric proportional hazards regression models, using an ordered multiple-failure stratified robust approach [37,38]. In addition to testing for difference, testing for equivalence of the two trial arms was done using the Westlake approach [39] with a dissimilarity significance cut-off of 5%. Model fit and diagnostics were also run to ensure no violation of the proportional hazards assumption occurred.

## Results

Between January 2005 and August 2008, 1072 pregnant women enrolled, 345 in Burkina Faso, 441 in Kenya and 284 in South Africa. Two women were excluded: one from Cohort 1 (abortion) and another from Cohort 2 (maternal death prior to delivery). Of the remaining, 118 initiated long-term antiretroviral treatment (Cohort 1; 118/1070; 11.0%) and 128 women received ZDV plus sdNVP in Cohort 2 (128/1070; 12.0%). The rest were randomized to triple ARV prophylaxis (RCT arm A, N = 412) or ZDV plus sdNVP (RCT arm B, N = 412). Eight women stopped or changed treatment due to anaemia (5 in Cohort 1, 3 in RCT arm A).

### Population description

Median age of participants ranged between 26 and 28 years across observation cohorts and trial arms (Table 1) and was similar across countries. Participants in South Africa had a higher SES than in Burkina Faso and Kenya (*P*-value < 0.001) with 57.4% (N = 284) of South Africans in the wealthiest SES category compared to only 6.4% (N = 346) in Burkina Faso and 6.1% (N = 440) in Kenya. SES was thus lower in the cohorts than RCT arms, given the absence of South African cohorts. Previous pregnancy was more common in Cohort 1 than 2, or the trial arms.

**Table 1:** Baseline characteristics of HIV-1 infected women enrolled in the Kesho Bora study, by intervention arm (N = 1070)

**Characteristic n (%)**	**Cohort 1:**	**Cohort 2:**	**RCT arm A:**	**RCT arm B:**
	**ART (CD4 < 200)**	**ZDV plus sdNVP (CD4 > 500)**	**Triple ARV (CD4 200–500)**	**ZDV plus sdNVP (CD4 200–500)**
	**(n = 118)**	**(n = 128)**	**(n = 412)**	**(n = 412)**
**Country**				
Burkina Faso	46 (39.0)	48 (38)	128 (31.1)	123 (29.9)
Kenya	72 (61.0)	80 (62)	141 (34.2)	148 (35.9)
South Africa	---	---	143 (34.7)	141 (34.2)
**Age** (years) median (IQR)	28 (26–31)	26 (23–29)	27 (24–31)	27 (23–31)
**Highest level of education attained**				
Never attended school	19 (16.1)	16 (12.5)	60 (14.6)	63 (15.3)
Primary	57 (48.3)	61 (47.7)	136 (33.0)	147 (35.7)
Secondary or higher	42 (35.6)	51 (39.8)	216 (52.4)	202 (49.0)
**WHO HIV clinical stage**				
1	28 (23.7)	100 (78.1)	279 (67.7)	296 (71.8)
2	41 (34.8)	23 (18.0)	109 (26.5)	93 (22.6)
3	25 (21.2)	5 (3.9)	24 (5.8)	23 (5.6)
4	24 (20.3)	0 (0)	0 (0)	0 (0)
**Nutritional status (BMI kg/m2)**				
Mean (SD)	25 (3.8)	26 (4.1)	27 (4.3)	27 (4.3)
Underweight: BMI < 18.5	3 (2.5)	1 (0.8)	1 (0.2)	2 (0.5)
**Socio-economic quintile**				
Most poor	30 (25.4)	32 (25.0)	66 (16.0)	81 (19.7)
Very poor	29 (24.6)	36 (28.1)	83 (20.1)	71 (17.2)
Poor	27 (22.9)	35 (27.3)	72 (17.5)	80 (19.4)
Less poor	25 (21.2)	16 (12.5)	96 (23.3)	79 (19.2)
Least poor	7 (5.9)	9 (7.0)	95 (23.1)	101 (24.5)
**Parity**				
Nulliparous (first pregnancy)	9 (7.6)	28 (21.9)	74 (18.0)	74 (18.0)
1	34 (28.8)	44 (34.4)	131 (31.8)	145 (35.2)
2	38 (32.2)	24 (18.7)	105 (25.5)	98 (23.8)
3 or more	37 (31.4)	32 (25.0)	102 (24.8)	95 (23.1)
**Planned mode of feeding**				
Breastfeeding	60 (50.9)	92 (71.9)	300 (72.8)	297 (72.1)
Replacement feeding	55 (46.6)	29 (22.6)	105 (25.5)	103 (25.0)
Unknown, undecided or other	3 (2.5)	7 (5.5)	7 (1.7)	12 (2.9)

*ART* = antiretroviral therapy; *triple ARV* = triple-combination antiretroviral prophylaxis; *ZDV plus sdNVP* = zidovudine plus single-dose nevirapine; *IQR* = interquartile range; *std dev* = standard deviation.

Women in Cohort 1 had more advanced HIV clinical disease, while the majority in Cohort 2 was asymptomatic (78%). CD4 cell count at enrolment was similar between the two RCT arms, but was higher in Arm A than B at childbirth and more than 150 cells higher at 6 months postpartum. At enrolment, the lowest mean body mass index (BMI) and highest proportion underweight (BMI < 18.5), was observed in Cohort 1. The RCT arms were balanced, with no differences detected in haematological or other measures at baseline.

About 75% of women in each trial arm and Cohort 2 initiated breastfeeding, compared to only 62% of those in Cohort 1 (Table 2). Duration of breastfeeding was, however, similar among those who did initiate breastfeeding in each group. As per study design, median duration of ARV use was 171.5 days in RCT arm A, compared with 65.5 days in RCT arm B (*P-*value *<* 0.001, Table 2), while women in Cohort 1 took ARV’s for a median 811.5 days. The median duration on ARV prior to childbirth in all arms was 5.0 weeks (Interquartile range [IQR] = 3.0-7.4), ranging from 5.4 weeks (IQR = 3.7-7.7) in Cohort 1, 4.6 weeks (IQR = 3.6-6.3) in Cohort 2, and 5.0 weeks in the RCT arms (IQR = 2.6-7.3 and 3.0-8.1 in RCT arm A and B respectively).

**Table 2:** Clinical and haematological findings of enrolled women, by intervention arm

**Characteristic**	**Cohort 1:**	**Cohort 2:**	**RCT arm A:**	**RCT arm B:**	***P*-value**^**i**^
	**ART (CD4 < 200)**	**ZDV plus sdNVP (CD4 > 500)**	**Triple ARV (CD4 200–500)**	**ZDV plus sdNVP (CD4 200–500)**	
	**(n visits = 1514)**	**(n visits = 1371)**	**(n visits = 4467)**	**(n visits = 4381)**	
Median weeks of antiretroviral use (IQR)	112.8	5.9	22.1	6.7	NA
	(85.1-117.7)	(4.7-6.8)	(10.6-32.4)	(4.6-8.9)	
Number of women who never initiated breastfeeding (formula only), n (%)	46 (38.9)	31 (24.2)	104 (25.2)	104 (25.2)	0.899*
Median weeks of breastfeeding if initiated (IQR)	19.1	17.9	20.9	19.6	0.898
	(11.9-23.2)	(12.1-24.7)	(11.9-24.9)	(11.3-25.0)	
Breastfeeding duration if initiated, n (%)					
<3 months	28 (43.8)	42 (46.1)	120 (41.2)	133 (44.8)	
≥3 months	36 (56.2)	49 (53.9)	171 (58.8)	164 (55.2)	0.385
Median CD4 count [cells/mm^3^] (IQR)					
At enrolment	134.5 (91–170)	621.5 (559–810)	335.5 (282–408)	338.5 (267–408)	0.428*
At delivery	184 (129–277)	742 (602.5-910)	462.5 (383–603)	415 (331–527)	<0.001*
At 6 months postpartum	274 (207–364)	731 (616–870)	479 (369–597)	374 (292–471)	<0.001*
At 12 months postpartum	304 (228–419)	683.5 (543–889)	401 (319.5-518)	378 (287–469)	0.509*
Median Hb [g/dL] (IQR)					
At enrolment	9.9 (8.9-11.0)	10.9 (9.9-12.7)	10.2 (9.2-11.1)	10.2 (9.1-11.1)	0.440*
At delivery	10.0 (8.9-11.8)	11.3 (10.2-13.3)	10.6 (9.6-11.6)	10.6 (9.4-11.8)	0.948*
At 6 months postpartum	11.8 (11.1-13.0)	13.1 (11.8-14.3)	11.9 (11.1-12.8)	11.7 (10.9-12.6)	0.075*
At 12 months postpartum	11.9 (11.0-12.9)	12.5 (11.4-13.6)	11.5 (10.7-12.5)	11.3 (10.3-12.2)	0.078*
Median MCV [fL] (IQR)					
At enrolment	86 (78–91)	85 (78–91)	87 (81–93)	88.5 (82–94)	0.866*
At delivery	91 (84–98)	88 (80–93)	91 (83–99)	93 (85–101)	0.325*
At 6 months postpartum	103 (96–110)	82 (77–90)	91 (83–100)	85 (79–90)	<0.001*
At 12 months postpartum	102 (95–110)	82 (75–89)	83 (77–89)	84 (78–90)	0.118*
Proportion with macrocytosis (95% CI)					
At enrolment	0.03 (0.01-0.08)	0.05 (0.02-0.10)	0.10 (0.07-0.13)	0.07 (0.05-0.10)	0.259**
At delivery	0.21 (0.14-0.30)	0.08 (0.04-0.15)	0.23 (0.18-0.27)	0.28 (0.24-0.33)	0.112*
At 6 months postpartum	0.63 (0.53-0.71)	0.01 (0.00-0.07)	0.26 (0.21-0.30)	0.03 (0.02-0.06)	<0.001*
At 12 months postpartum	0.61 (0.51-0.70)	0.01 (0.00-0.07)	0.06 (0.03-0.10)	0.09 (0.06-0.14)	0.144*
Proportion with any anaemia (Hb < 11 g/dL) (95% CI)					
At enrolment	0.74 (0.65-0.82)	0.55 (0.46-0.64)	0.71 (0.66-0.75)	0.72 (0.68-0.76)	0.627*
At delivery	0.71 (0.61-0.79)	0.43 (0.33-0.52)	0.61 (0.56-0.66)	0.59 (0.54-0.64)	0.561*
At 6 months postpartum	0.25 (0.18-0.34)	0.12 (0.06-0.2)	0.23 (0.19-0.28)	0.26 (0.21-0.30)	0.398
At 12 months postpartum	0.21 (0.14-0.30)	0.16 (0.09-0.25)	0.32 (0.26-0.38)	0.39 (0.34-0.45)	0.228*
Proportion with severe anaemia (Hb < 8 g/dL) (95% CI)					
At enrolment	0.09 (0.05-0.16)	0.04 (0.01-0.09)	0.06 (0.04-0.09)	0.07 (0.04-0.09)	0.671
At delivery	0.15 (0.09-0.23)	0.06 (0.02-0.12)	0.06 (0.04-0.09)	0.10 (0.07-0.13)	0.144*
At 6 months postpartum	0.02 (0–0.06)	0.01 (0.00-0.06)	0.01 (0.00-0.03)	0.02 (0.01-0.04)	0.255
At 12 months postpartum	0.01 (0–0.04)	0.00 (0.00-0.04)	0.02 (0.00-0.08)	0.03 (0.01-0.09)	0.678
Cumulative incidence of severe anaemia (Hb < 8 g/dL) (95% CI)					
At delivery	0.14 (0.09-0.22)	0.05 (0.03-0.11)	0.09 (0.06-0.12)	0.08 (0.06-0.11)	0.512*
At 6 months postpartum	0.30 (0.23-0.39)	0.10 (0.06-0.16)	0.16 (0.13-0.20)	0.17 (0.14-0.21)	0.436*
At 12 months postpartum	0.33 (0.26-0.41)	0.11 (0.06-0.17)	0.18 (0.14-0.21)	0.19 (0.16-0.23)	0.712*
At 18 months postpartum	0.34 (0.27-0.42)	0.11 (0.06-0.17)	0.18 (0.15-0.22)	0.21 (0.17-0.25)	0.356*
Overall incidence of severe anaemia per person year (Hb < 8 g/dL) (95% CI)	0.21	0.06	0.12	0.14	0.317*
	(0.16-0.27)	(0.04-0.10)	(0.10-0.15)	(0.12-0.18)	
Number of single failures only (Hb < 8 g/dL), n (%)	28 (23.7)	11 (8.6)	49 (11.9)	65 (15.8)	0.129*
Number of individuals with repeat failures (Hb < 8 g/dL), n (%)	11 (9.3)	2 (1.6)	14 (3.6)	14 (3.3)	0.708*
Summed ^ii^ number of repeat failures, n	24	4	35	32	---

*CI* = confidence interval; *IQR* inter-quartile range; *SD* = standard deviation; *Hb* = haemoglobin; *ART* = antiretroviral therapy; *triple ARV* = triple-antiretroviral prophylaxis; *ZDV plus sdNVP* = zidovudine plus single-dose nevirapine; i: comparing RCT arms only: χ2 (or Fishers Exact) test for categorical variables, Student's *t* test for comparison of two means or ANOVA for comparison of multiple means, Rank Sum test if data were highly skewed, and log-rank test for equality of survivor functions; ii: individuals x number of failures; *P-value <0.05 based on comparison of all 4 groups; **p-value between 0.05 and 0.10 in comparison of all 4 groups.

### Haematological measures

At enrolment, mean Hb was 10.3 g/dL (standard deviation [SD] = 1.7 g/dL) and 69.9% had anaemia (<11.0 g/dL), highest in Burkina Faso (79.3%) followed by Kenya (70.3%) and South Africa (57.8%; *P*-value < 0.001). Mean Hb in the lowest SES quintile was 10.1 g/dL (SD = 1.9) at enrolment compared to 10.7 (SD = 1.6) in the wealthiest quintile (*P-*value < 0.001). Though mean Hb in Cohort 1 (10.1) was a full 1 g/dL lower than in Cohort 2 at enrolment (11.2, *P-*value < 0.001), by 6 months postpartum, no differences were detected between mean Hb in these two groups and the average Hb was similar at month 12.

In Cohort 1, the proportion of women with macrocytosis increased to nearly two-thirds at months 6 and 12 (Table 2). At 6 months postpartum, mean MCV levels were significantly higher in RCT arm A than arm B (91 vs 85 fl), but differences were not detected between trial arms at other time points. However, the proportion with macrocytosis decreases significantly within 3 months after treatment cessation in both RCT arms i.e. by month 3 post childbirth for RCT arm B and by month 9 post childbirth for RCT arm A, as per design.

Across all study groups, a rapid and substantial increase in Hb was observed after initiation of ARVs especially following childbirth (*P*-value *<* 0.001 in each study group using repeated measures ANOVA assessing mean change at multiple time points). Increases in Hb in each of the time intervals reached a plateau of 1.5-2.0 g/dL median rise in Hb compared to enrolment levels (Figure 1). In Cohort 2, however, we observed a subsequent decrease in Hb following ARV cessation, though this group did have a higher median enrolment Hb compared to the other arms and attained higher median gains within the first 3 months of follow-up compared to the RCT arms (Figure 1). The median rise (and plateau) in Hb in the two trial arms was very similar with minor non-statistically significant differences (Table 2). Both arms appeared to maintain these levels through the follow-up period (Figure 1).

**Figure 4:**
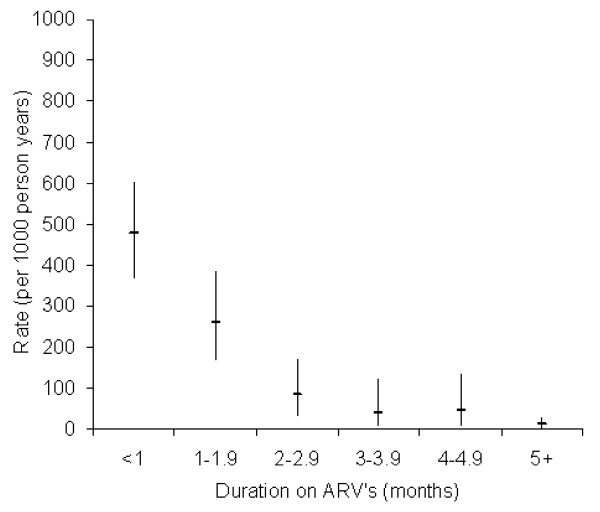
**Incidence of severe anaemia (including 95% confidence intervals) by ARV duration for all study arms (includes delivery related failures occurring at median t = 1.15 months following treatment initiation) (*P-*** **value comparing incidence in <1 versus 1–1.9 month duration = 0.004).**

The highest period prevalence of severe anaemia in the period between enrolment and delivery was observed in Kenya (8.4%, *P*-value *<* 0.001). Kaplan-Meier failure curves for severe anaemia by intervention arm suggests that most failures occurred within the first two months after initiation of ARV drugs (Figure 2), which corresponds largely with the period between enrolment and childbirth. We observed a strong reduction in severe anaemia incidence with increasing duration of receiving ARV (Figure 4) – further confirmed in the multivariate analysis below. More than one month on ARV’s was associated with a marked decrease in severe anaemia risk when compared to under one month on ARV’s (i.e. same individuals either side of this duration cut-off). Longer ARV duration (two or more months) further significantly reduced anaemia incidence. We observed 64 cases of anaemia among breastfeeding women at a rate of 54 per 1000 person-years (95% CI = 42-69) compared to 24 cases or 52 per 1000 (95% CI = 35-77) among women who did not breastfeed at all (log rank *P*-value = 0.830).

A log rank test comparing all four groups indicated a highly significant difference in failure functions (Figure 2), irrespective of using date of enrolment or delivery as commencement of risk. The highest incidence of severe anaemia was observed in Cohort 1 and lowest in Cohort 2. The cumulative incidence proportion of severe anaemia at 18 months postpartum was highest in Cohort 1 (0.34) followed by RCT arm B (0.21), RCT arm A (0.18) and lowest in Cohort 2 (0.11; Table 2). No difference was detected in the failure function of the two RCT arms (log-rank *P*-value = 0.344). Based on equivalence testing, dissimilarity could also not be rejected i.e. RCT arms were equivalent (*P*-value = 0.323). Kaplan-Meier analysis of severe anaemia failure in the RCT arms, stratified by enrolment Hb level, showed a higher risk of severe anaemia failure in the group that had mild anaemia at enrolment (Hb 8.0-11.0 g/dL), compared to those with no anaemia at enrolment (Hb >11.0 g/dL) (log rank *P*-value *<* 0.001; Figure 3). No difference (log rank *P*-value = 0.587) was detected in severe anaemia failure in the RCT arms comparing those who had mild anaemia at enrolment. However, despite small numbers, higher failure (*P*-value = 0.026) was observed in RCT arm B than in RCT arm A in women with enrolment Hb > 11.0 g/dL (Figure 3), though this does not adjust for explanatory or confounding factors.

In multivariable analysis of the observational cohorts, increasing duration on ARV was found to be highly protective in cohort 1 (hazard ratio (HR) = 0.04; *P*-value = 0.002) while the lowest SES group had a 4.1 higher hazard ratio (HR) of severe anaemia compared to the highest SES group in Cohort 2 (95% CI = 1.5-10.8; Table 3). In the RCT trial arms, maternal age and parity were not associated with severe anaemia in bivariate analysis (*P*-value ≥ 0.2). Trial arm was forced into the final RCT model, even though it was not associated with severe anaemia in bivariate analysis (HR = 1.09, 95% CI = 0.77-1.52) and remained non-significant following multivariable adjustment. The following factors were associated with increased severe anaemia failure in the RCT arms following multivariable adjustment: Kenyan site (HR = 2.76, 95% CI = 1.79-4.25) using Burkina Faso as reference, advanced HIV disease (HR = 2.11, 95% CI = 1.19-3.75) and prolonged breastfeeding (> = 6 months) (HR = 1.62, 95% CI = 1.00-2.62). The following factors were associated with reduced risk following multivariable adjustment: longer cumulative duration of ARV (HR = 0.05, *P*-value = 0.003), and marginally the South African site (HR = 0.52, *P*-value = 0.054).

**Table 3:** Factors associated with severe anaemia (Hb <8.0 g/dL) using Multivariable Cox models by cohort and RCT arm

		**Cohort 1**			**Cohort 2**			**RCT arms**		
**Predictor**	**Anaemia failures/n**	**Unadjusted hazard ratio (HR)**	**Adjusted HR**	***P*-value**^**ii**^	**Unadjusted HR**	**Adjusted HR**	***P*-value**^**ii**^	**Unadjusted HR**	**Adjusted HR**	***P*-value**^**ii**^
		**(95% CI)**	**(95% CI) **^**i**^		**(95% CI)**	**(95% CI) **^**i**^		**(95% CI)**	**(95% CI) **^**i**^	
Observational cohort										
Cohort 1: ART (CD4 < 200)	52/118	---	---	---	---	---	---	---	---	---
Cohort 2: ZDV plus sdNVP (CD4 > 500)	15/128	---	---	---	---	---	---	---	---	---
Intervention arm^iii^										
RCT arm A: Triple ARV (CD4 200–500)^iv/v^	97/412	---	---	---	---	---	---	1	1	---
RCT arm B: ZDV plus sdNVP (CD4 200–500)^iv/v^	84/412	---	---	---	---	---	---	1.09 (0.77,1.52)	0.78 (0.54,1.11)	0.166
Country										
Burkina Faso	70/345	**1**	1	---	1	---	---	1	1	---
Kenya	157/441	**1.66 (0.88,3.13)**	1.56 (0.80,3.07)	0.192	1.11 (0.41,3.02)	---	---	**2.70 (1.76,4.13)**	**2.76 (1.79,4.25)**	**<0.001**
South Africa	21/281	---	---	---	---	---	---	**0.42 (0.23,0.75)**	**0.52 (0.26,1.01)**	**0.054**
Age (enrolment)	248/1070	**0.95 (0.88,1.02)**	0.96 (0.89,1.04)	0.312	0.95 (0.82,1.11)	---	---	1.00 (0.97,1.04)	---	---
BMI (enrolment)	248/1070	1.02 (0.93,1.11)	---	---	0.97 (0.87,1.09)	---	---	**0.96 (0.92,1.01)**	1.00 (0.95,1.05)	0.964
Secondary or higher education versus none or primary level (enrolment)	90/511		---	---		---	---	**0.57 (0.40,0.81)**	0.85 (0.56,1.26)	0.413
HIV stage 3 ^vi^ versus stage 1 or 2 (enrolment)	48/101	1.31 (0.71,2.44)	---	---	Insufficient observations	---	---	**2.11 (1.18,3.79)**	**2.11 (1.19,3.75)**	**0.011**
Most poor SES category versus poor or least poor (enrolment)	73/209	1.44 (0.73,2.85)	---	---	**5.13 (1.83,14.41)**	**4.08 (1.54,10.75)**	**0.005**	**1.57 (1.06,2.32)**	1.13 (0.75,1.70)	0.567
First pregnancy (enrolment)	37/185	0.90 (0.34,2.41)	---	---	1.35 (0.44,4.19)	---	---	0.88 (0.56,1.40)	---	---
Never breastfed	69/285	1.49 (0.80,2.78)	---	---	0.24 (0.03,1.86)	---	---	1.00 (0.66,1.50)	---	---
Prolonged breastfeeding										
< 6 months	138/640	1	1	---	1	1	---	1	1	---
> = 6 months	31/103	0.79 (0.28,2.23)	---	---	0.77 (0.19,3.13)	1.12 (0.27,4.76)	0.873	**1.46 (0.88,2.41)**	**1.62 (1.00,2.62)**	**0.051**
0 duration (did not breast-feed)	69/285	1.43 (0.74,2.79)	**---**	**---**	0.23 (0.03,1.80)	0.33 (0.04,2.48)	0.280	1.06 (0.69,1.61)	1.05 (0.68,1.62)	0.831
Cumulative duration on ARV (in years, continuous scale)	248/1070	**0.03 (0.00,0.27)**	**0.04 (0.00,0.29)**	**0.002**	0.02 (0.00,vii)	---	---	**0.03 (0.00,0.24)**	**0.05 (0.01,0.37)**	**0.003**

*CI* = confidence interval; *BMI* = body mass index; SES = socio-economic status.

i: Test of proportional hazards assumption not violated for cohort specific and RCT models.

ii: log rank test.

iii: *ART* = antiretroviral therapy; triple ARV = triple-combination antiretroviral prophylaxis; ZDV plus sdNVP = zidovudine plus single-dose nevirapine.

iv: difference test result comparing the two RCT trial arms i.e. null that difference = 0 (*P*-value = 0.636).

v: equivalence test results comparing the two RCT trial arms (*P*-value = 0.323; null hypothesis of dissimilarity rejected at pre-specified level of 5%, and equivalence assumed).

vi: no participant s in the RCT arms with stage 4 at enrolment.

vii: small sample size (*P*-value = 0.567).

## Discussion

The evidence from this trial shows the safety and efficacy of combination antiretroviral drugs during pregnancy, delivery and breastfeeding and supports the revised 2010 WHO guidelines [29].

While previous evidence indicates that ZDV may be cytotoxic, so is HIV infection. The potential negative consequences of longer durations of ZDV exposure are seemingly ameliorated by the marked impact of triple ARV drugs on HIV disease [29], evidenced by the rise in CD4 cell count in RCT arm A, to levels around 150 cells higher than RCT arm B at six months postpartum. This group also had higher haemoglobin levels postpartum than RCT arm B and lower rates of failure in those without pre-existing anaemia. However, we did observe a higher proportion of macrocytosis in RCT arm A compared to B at 6 months follow-up. Improvements in Hb in Cohort 1, which approximated levels in Cohort 2 by 12 months postpartum, further demonstrates the positive effects of triple-ARV regimens on maternal Hb, which compensate for any haemosuppressive effects of ZDV. Overall, the prevalence of anaemia (Hb < 11 g/dL) in this multi-country study population ranged between 55-74%, and are similar to levels in other African settings [3-6]. This is a very important condition, given its links with maternal morbidity and mortality, especially among HIV-positive women [40,41].

### Association between ARV use during pregnancy and severe anaemia

A pronounced increase in Hb was observed in all groups following initiation of antiretroviral drugs, particularly post-childbirth. This phenomenon has been documented in other randomized trials in treatment-naïve patients (pregnancy was not an exclusion criteria in a meta-analysis of 6 RCTs) [42]. All treatment groups displayed an increase in Hb to reach a plateau gain of +1.5-2 g/dL. Following treatment cessation in Cohort 2 (enrolment CD4 > 500) we observed a decline in Hb, while the gains made were maintained long term in the RCT groups and extended in Cohort 1. The Hb decline in Cohort 2 appears to be more of a decline of Hb with progression of HIV disease, given that they started the trial with higher CD4 and Hb levels compared to the other groups. The impact of the maternal package at enrolment (iron and folic acid supplementation, presumptive treatment for malaria and anti-helminthics) may also have contributed to the rise in Hb.

No differences were detected between the two RCT intervention arms in occurrence of severe anaemia following multivariable adjustment and tests suggested equivalency.

Berhane et al. found that increased duration of ARV use among women (mixed cohort of non-pregnant and pregnant women [43]) resolved pre-existing anaemia after ARV use for at least 6 months and reduced the development of anaemia after 12 or more months of treatment [40]. This association of ARV with improvement of the anaemia has also been reported elsewhere [44]. Our findings suggest that a significant reduction in severe anaemia risk among pregnant women already exists after one month use of ARV and suggests significant clinical implications. Longer cumulative duration of ARV use was also an important predictor of reduced severe anaemia risk in the RCT trial arms and in cohort 1, even after multivariable adjustment. A long term residual protection following prophylaxis cessation was also evident as observed by the maintenance of Hb levels post treatment in the RCT arms. This study thus provides further empirical evidence to reassure policy makers about the safety of longer durations of exposure to ZDV-containing triple prophylaxis compared to a shorter ZDV-only regimen, especially concerning the suspected haematological effects of ZDV-containing prophylaxis taken for longer periods or during lactation. Moreover, overall evidence is also reassuring about the effects of combined ARV drugs on pregnancy outcomes other than anaemia [26,29].

### Factors associated with development of severe anaemia

Among HIV-positive observational cohort women who were free of severe anaemia at baseline, only SES category was independently associated with severe anaemia risk. Conversely several factors were associated with severe anaemia in the RCT arms following multivariable adjustment. These factors are consistent with those identified in previous studies [9,40,45-47]. These included local setting (country), more advanced HIV disease, prolonged breastfeeding and duration of ARV. The observed reduced anaemia risk in South African and converse elevated risk in Kenya compared to Burkina Faso, is likely explained by the fact that little or no malaria exists in the study sites in South Africa while malaria-associated anaemia is common in the Kenyan site [48]. Nutritional differences between study sites also cannot be discounted.

We did not observe a significant difference in severe anaemia incidence when comparing women who did not breastfeed to those who did. Notably however, breastfeeding more than 6 months was associated with an increased risk of anaemia in the trial arms of the study population. Current WHO guidelines promote exclusive breastfeeding for the first 6 months for women on ARV. These guidelines suggest the introduction of an appropriate complementary food thereafter, and continued breastfeeding for 12 months. The finding of raised anaemia risk may have implications for future decisions about recommended duration of breastfeeding in HIV-positive women, and should be further researched [9].

### Study limitations

These data were analysed from a completed trial that was not specifically designed to assess this secondary outcome. Higher levels of care received by participants may also limit the generalisability of study findings. Women had more visits to antenatal clinics than those in the general population, and thus likely received more iron and vitamin supplementation, and adherence counselling. Further, the study population was predominately urban and may not be representative of rural women. As women with severe anaemia were excluded from study participation, we were unable to assess the effects of zidovudine in this small group. Most previous PMTCT studies similarly exclude women with severe anaemia at enrolment [21-24]. We also included unscheduled visits and this may have influenced the results as sicker women might have received additional Hb screening measures.

## Conclusions

This study demonstrates that similar severe anaemia risk exists when comparing the short and longer ZDV-containing regimens. This should reassure policy makers who are increasingly recommending longer ARV regimens for PMTCT in low- and middle-income countries. Until 2010, based on concerns around drug safety, among other considerations, many of these countries used much shorter regimens. The longer the period that ARV’s are taken in pregnancy or breastfeeding, the lower the risk of HIV in the infant. ARV duration is the most critical factor in determining whether children acquire HIV. The study also confirms the importance of key independent predictors of anaemia, showing the groups that require closer monitoring. Finally, ARV use for as little as one month may be beneficial to reduce severe anaemia risk among pregnant (and postpartum) women and may provide long term protection following cessation of prophylaxis.

## Endnote

^a^Percentage of the volume of whole blood that is made up of red blood cells, normally ~40% in women.

## Appendix 1: the Kesho Bora Study Group and acknowledgements

### Study sites

(1) Bobo Dioulasso, Burkina Faso (Centre Muraz): Nicolas Meda (Principal Investigator), Paulin Fao, Odette Ky-Zerbo, Clarisse Gouem (Study coordinators), Paulin Somda, Hervé Hien, Patrice Elysée Ouedraogo, Dramane Kania, Armande Sanou, Ida Ayassou Kossiwavi, Bintou Sanogo, Moussa Ouedraogo, Issa Siribie (Investigators), Diane Valéa (Laboratory Coordinator), Sayouba Ouedraogo & Roseline Somé (Data Managers), François Rouet (Inter-Site Laboratory Coordination).

(2) Durban, South Africa (University of KwaZulu Natal): Nigel Rollins (Principal Investigator), Lynne McFetridge, Kevi Naidu (Study Coordinators).

(3) Mombasa, Kenya (International Centre for Reproductive Health): Stanley Luchters, Marcel Reyners (Principal Investigators), Eunice Irungu (Study Coordinator), Christine Katingima, Mary Mwaura and Gina Ouattara (Investigators), Kishor Mandaliya, Sammy Wambua (Laboratory Coordinators), Mary Thiongo (Data Manager).

(4) Nairobi, Kenya (Network for AIDS Researchers in East and Southern Africa): Ruth Nduati (Principal Investigator), Judith Kose (Study Coordinator), Ephantus Njagi (Laboratory Coordinator), Peter Mwaura (Data Manager).

(5) Somkhele, South Africa (Africa Centre for Health and Population Studies, University of KwaZulu Natal): Marie-Louise Newell (Principal Investigator), Stephen Mepham (Study Coordinator), Johannes Viljoen (Laboratory Coordinator), Ruth Bland (Investigator), Londiwe Mthethwa (Data Manager).

### Supporting institutions

(1) Agence Nationale de Recherches sur les SIDA et les Hépatites Virales, France: Brigitte Bazin, Claire Rekacewicz (Sponsor Representatives).

(2) Centers for Disease Control and Prevention, USA: Allan Taylor (Sponsor Representative and Co-Investigator), Nicole Flowers, Michael Thigpen, Mary Glenn Fowler, Denise Jamieson (Co-Investigators).

(3) *Eunice Kennedy Shriver* National Institute of Child Health and Human Development, National Institutes of Health, USA: Lynne M. Mofenson (Sponsor Representative), Jennifer S. Read (Co-Investigator).

(4) Institut de Recherche pour le Développement (IRD), Montpellier, France: Kirsten Bork, Cécile Cames and Amandine Cournil (Nutrition Coordination).

(5) International Centre for Reproductive Health (ICRH), Ghent University, Ghent, Belgium: Patricia Claeys, Marleen Temmerman, Stanley Luchters (Sponsor Representatives).

(6) Université Montpellier 1, EA 4205 “Transmission, Pathogenèse et Prévention de l’infection par le VIH » ; and CHU Montpellier, Laboratoire de Bactériologie-Virologie, Montpellier, France: Philippe Van de Perre, Pierre Becquart (until December 2006), Vincent Foulongne, Michel Segondy (Laboratory Coordination).

### Study coordination

World Health Organization, Geneva, Switzerland: Isabelle de Vincenzi (Study Coordinator), Philippe Gaillard (Site Coordinator), Tim Farley (Project Manager), Ndema Habib (Study Statistician), Sihem Landoulsi (Study Analyst).

### Funding

The Bobo-Dioulasso site was funded by l'Agence Nationale de Recherches sur le Sida et les Hépatites Virales (ANRS) and UNDP/UNFPA/World Bank/WHO Special Programme of Research, Development and Research Training in Human Reproduction (WHO/HRP).

The Mombasa site was funded by ANRS, WHO/HRP, European and Developing Countries Clinical Trials Partnership (EDCTP), Thrasher Research Fund, Belgian Directorate General for International Cooperation.

The Nairobi site was funded by the Centers for Disease Control and Prevention (CDC) and the *Eunice Kennedy Shriver* National Institute of Child Health and Human Development (NICHD) through a cooperative agreement.

The South-African sites were funded by the Department for International Development (DFID), EDCTP, UNICEF and WHO/HRP.

The Nutrition and laboratory coordination were funded by ANRS.

The overall coordination and external monitoring was funded by WHO/HRP.

Representatives of ANRS, CDC, NICHD and WHO/HRP were involved in study design and collection, analysis and interpretation of data.

The findings and conclusions in this report are those of the authors and do not necessarily represent the views of the World Health Organization, of the Centers for Disease Control and Prevention or of the National Institutes of Health.

The authors gratefully acknowledge the contribution to this work of the Victorian Operational Infrastructure Support Program received by the Burnet Institute.

## Abbreviations

ANOVA: Analysis of variance; ARV: Antiretroviral; BMI: Body mass index; CI: Confidence interval; Hb: Haemoglobin; HCT: Haematocrit; HR: Hazard ratio; HIV: Human immunodeficiency virus; IQR: Interquartile range; 3TC: Lamivudine; MCH: Mean cell Hb; MCV: Mean cell volume; PMTCT: Preventing mother-to-child transmission; RCT: Randomized controlled trial; sd-NVP: Single-dose nevirapine; SES: Socio-economic status; SD: Standard deviation; SSA: Sub-Saharan Africa; ZDV: Zidovudine.

## Competing interests

The authors declare that they have no competing interests.

## Authors’ contributions

MM, NM, MT, MLN, TF, SL and the Kesho Bora Study Group were involved in the conception, design and/or supervision of the primary study. MC, TF and SL conceptualised this sub-analysis. BS analysed the data. BS, MC and SL interpreted the data, and BS drafted the manuscript. All authors critically reviewed the manuscript and approved the final manuscript version for publication.

## Pre-publication history

The pre-publication history for this paper can be accessed here:

http://www.biomedcentral.com/1471-2334/13/522/prepub
